# Whole-Genome Sequences of Four *Salmonella enterica* Serotype Newport Strains from Humans

**DOI:** 10.1128/genomeA.00213-13

**Published:** 2013-05-09

**Authors:** Jianmin Zhang, Guojie Cao, Xuebin Xu, Huiming Jin, Qiong Zhang, Jianwei Chen, Xiaowei Yang, Haijian Pan, Xiuli Zhang, Marc Allard, Eric Brown, Jianghong Meng

**Affiliations:** Shanghai Jiao Tong University, Shanghai, Chinaa;; Department of Nutrition and Food Science, University of Maryland, College Park, Maryland, USAb;; Joint Institute for Food Safety and Applied Nutrition (JIFSAN), University of Maryland, College Park, Maryland, USAc;; Shanghai Municipal Center for Disease Control and Prevention, Shanghai, Chinad;; BGI-Shenzhen, Shenzhen, Chinae;; Henan Center for Disease Control and Prevention, Henan, Chinaf;; Division of Microbiology, Center for Food Safety and Applied Nutrition, Food and Drug Administration, College Park, Maryland, USAg

## Abstract

Salmonellosis contributes significantly to the public health burden globally. *Salmonella enterica* serotype Newport is among *Salmonella* serotypes most associated with food-borne illness in the United States and China. It was thought to be polyphyletic and to contain different lineages. We report draft genomes of four *S*. Newport strains isolated from humans in China.

## GENOME ANNOUNCEMENT

Nontyphoidal *Salmonella* causes an estimated 93.8 million cases of gastroenteritis and 155,000 deaths each year in the world (1). *Salmonella enterica* serotype Newport ranks among the top three serotypes associated with food-borne illness in the United States, causing at least 100,000 infections annually (2). In China, *S*. Newport is also among the most common serotypes isolated from patients with diarrhea (3). The serotype was thought to be polyphyletic, with extensive genomic diversity, containing three independent lineages (4, 5).

Currently, a total of 32 completed genomes and 277 draft genomes of *Salmonella* have been deposited in GenBank, among which there were one completed genome and 27 draft genomes of *S*. Newport. Two of the genomes were from *S*. Newport strains isolated from clinical sources. In the present report, we announce the availability of four draft genomes of *S*. Newport strains from stool specimens of patients with diarrhea in China: SH111077, Shandong_3, Henan_3, and JS09102. The genome data provide insights on the genomic diversity and evolutionary history of *S.* Newport.

The four *S*. Newport strains were sequenced using the HiSeq 2000 platform (Illumina, San Diego, CA) to obtain 39 to 43× coverage draft genomes. Genomic data were assembled with SOAPdenovo 1.05 (http://soap.genomics.org.cn/soapdenovo.html). The data of each draft genome are as follows: SH111077 (80 contigs longer than 500 bp, genome size of 4,868,771 bp, and contig N_50_ of 131,989), Shandong_3 (55 contigs longer than 500 bp, genome size of 4,752,037 bp, and contig N_50_ of 194,658), Henan_3 (68 contigs longer than 500 bp, genome size of 4,812,853 bp, and contig N_50_ of 183,174), and JS09102 (80 contigs longer than 500 bp, genome size of 5,078,742 bp, and contig N_50_ size of 160,516). Sequences were annotated with the NCBI Prokaryotic Genomes Automatic Annotation Pipeline (6).

### Nucleotide sequence accession numbers.

The accession numbers of the four *S.* Newport genome sequences in GenBank are AOGJ00000000, AOGI00000000, AOGH00000000, and AOGG00000000.
